# Chondrocyte Deformations as a Function of Tibiofemoral Joint Loading Predicted by a Generalized High-Throughput Pipeline of Multi-Scale Simulations

**DOI:** 10.1371/journal.pone.0037538

**Published:** 2012-05-23

**Authors:** Scott C. Sibole, Ahmet Erdemir

**Affiliations:** Computational Biomodeling (CoBi) Core and Department of Biomedical Engineering Lerner Research Institute, Cleveland Clinic, Cleveland, Ohio, United States of America; Cleveland Clinic, United States of America

## Abstract

Cells of the musculoskeletal system are known to respond to mechanical loading and chondrocytes within the cartilage are not an exception. However, understanding how joint level loads relate to cell level deformations, e.g. in the cartilage, is not a straightforward task. In this study, a multi-scale analysis pipeline was implemented to post-process the results of a macro-scale finite element (FE) tibiofemoral joint model to provide joint mechanics based displacement boundary conditions to micro-scale cellular FE models of the cartilage, for the purpose of characterizing chondrocyte deformations in relation to tibiofemoral joint loading. It was possible to identify the load distribution within the knee among its tissue structures and ultimately within the cartilage among its extracellular matrix, pericellular environment and resident chondrocytes. Various cellular deformation metrics (aspect ratio change, volumetric strain, cellular effective strain and maximum shear strain) were calculated. To illustrate further utility of this multi-scale modeling pipeline, two micro-scale cartilage constructs were considered: an idealized single cell at the centroid of a 100×100×100 μm block commonly used in past research studies, and an anatomically based (11 cell model of the same volume) representation of the middle zone of tibiofemoral cartilage. In both cases, chondrocytes experienced amplified deformations compared to those at the macro-scale, predicted by simulating one body weight compressive loading on the tibiofemoral joint. In the 11 cell case, all cells experienced less deformation than the single cell case, and also exhibited a larger variance in deformation compared to other cells residing in the same block. The coupling method proved to be highly scalable due to micro-scale model independence that allowed for exploitation of distributed memory computing architecture. The method’s generalized nature also allows for substitution of any macro-scale and/or micro-scale model providing application for other multi-scale continuum mechanics problems.

## Introduction

The phenomenon of cell behavior being directed by mechanical stimuli, referred to as mechanotransduction or mechanoregulation, as well as cell damage resulting from mechanical disruption, have been a topic of research in medicine and biology for several decades [1]–[3]. Often, research has been conducted at the spatial scale of the cell and its immediate extracellular environment. While cellular activity at the micro-scale alters the mechanical environment, loading transferred from higher spatial scales also plays a role [4]. For this reason, the search for a better understanding of multi-scale spatial interactions has become an increasingly desirable objective, in order to establish the causal mechanical relationships between the loading of joints, tissues, and cells.

In the field of biomechanics, a strong motivation to better understand the mechanics of articular cartilage exists [5]. This is due to the high prevalence of pathologies such as osteoarthritis (OA), which affects approximately 27 million adults in the United States [6] and can drastically reduce quality of life. The progression of OA exhibits changes in tissue structure [7]–[9], as well as changes in cellular (chondrocyte) distribution and behavior [10]–[14]. While these changes occur at the tissue or cellular scales, a general consensus exists that the loading of the joints likely plays a role in their onset. Even in the absence of pathology, cartilage may experience changes simply as a result of aging. As with OA, these changes occur at the tissue [15], [16] and cellular scale [17], [18]. In addition, modifications of joint-scale mechanics also occur with aging [19]–[21]. Clearly, cartilage mechanics is a multi-scale paradigm and a means to investigate the interplay between scales is crucial to extending the understanding of the *in vivo* function of this biological material.

While pathology and aging impact cartilage mechanics at multiple scales, before addressing such complexities, understanding the normal joint, tissue, and cellular mechanical behavior is necessary. Experimental acquisition of mechanical data, while attainable in animal studies [22], becomes more difficult as the spatial scale decreases. If one attempts to gather data at different spatial scales simultaneously under lifelike loading scenarios, e.g. synchronous measurement of joint level kinematics and/or kinetics and cell deformation, the present technology is inadequate. Computational modeling techniques provide an avenue to obtain additional insights about mechanics at various spatial scales. Further, passing information between models at different spatial scales allows for investigation of load transfer mechanisms.

Extensive research utilizing computational modeling, particularly finite element analysis (FEA), has been conducted at each of these individual spatial scales. At the tissue and cellular scale, complex constitutive models have been applied to simplified geometric and loading cases [23]. In contrast, at the joint scale, simpler material models were typically employed, allowing representation of complex geometry and physiological loading conditions to be considered [24]. Ultimately, relating models at both scales will provide insights about the mechanics a chondrocyte experiences *in vivo*.

An array of approaches can be taken to understand the mechanical coupling between joint function and chondrocyte response. Explicitly modeling the micro-scale components in the macro-scale model constitutes the method with the least abstraction. This approach is often used in the modeling of cancellous bone [25], but requires a tremendous finite element count for even small volumes. For the dimensions and added complexities of a joint, the computational cost is too excessive given current technology.

Computational homogenization, in which the material behavior at the macro-scale at each non-linear solution step is a result of the solution of a micro-scale model at every integration point in the macro-scale mesh, provides another avenue for relating spatial scales [26]. While still very computationally expensive, solutions can be obtained with this method [27], but for advanced problems, require access to large shared-memory architectures which are less common than distributed-memory platforms. It should also be noted that robust simulations using this method may not be possible since the failure of a single micro-scale model to converge, if not contained, results in the failure of the entire macro-scale solution.

Finally, a simple post-processing method can be employed. This involves first obtaining a solution of the macro-scale model, possibly using FEA. The deformation experienced by each finite element in this model can then be used to generate boundary conditions for an array of micro-scale models, which are then solved to determine the micro-scale response. While this provides a weaker coupling between scales than the previously described methods, and therefore must satisfy multiple assumptions about consistency between scales, it provides a unique advantage in that all models are independent or autonomous. This allows for exploitation of distributed-memory architectures which can provide tremendous computing power, since there is no need to communicate information between micro-scale models. Further, this approach is more robust in that, failure of a micro-scale model does not result in failure of the entire process. While the macro-scale mechanics are not a direct function of micro-scale response (as in computational homogenization), a post-processing pipeline would provide a cost-effective platform for descriptive analysis of cell deformations under different joint loading.

The post-processing approach, to relate joint mechanics to cell mechanics, was used to achieve the three-fold objective in this study: i) to establish a pipeline to post-process macro-scale finite element analysis results to estimate micro-scale cell deformations for desired macro-scale regions at a desired time point of macro-scale loading, ii) to illustrate the pipeline’s utility through estimation of cell deformations in middle-zone cartilage for compressive loading of the tibiofemoral joint, and iii) to explore the differences in the results of multi-scale modeling of state-of-the-art and anatomically-based assumptions of cell distribution in a micro-scale volume.

## Methods

**Figure 1:**
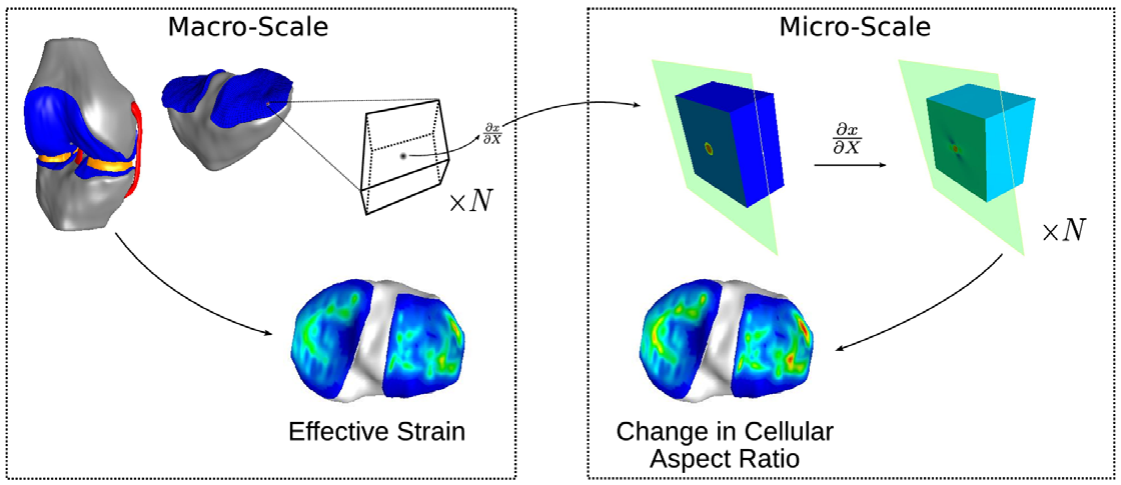
The autonomous approach to couple joint, tissue, and cell scales is shown above. Left: The method began with the FEA of a macro-scale model for a kinetic/kinematic scenario. Deformation gradients at the centroids of finite elements in tissue regions of interest were calculated from element nodal positions. Right: Displacement boundary conditions were prescribed to surface nodes of independent micro-scale models corresponding to the deformation gradient of each finite element experiencing deformation in tissue regions considered. Results of micro-scale FEA solutions were post-processed to calculate deformation metrics of interest.

The autonomous approach implemented in this study, involved a series of stages beginning with the FEA of a single macro-scale model at the joint scale, calculation of deformation gradients at element centroids, using these deformation gradients to prescribe boundary conditions for micro-scale models for each macro-scale element of interest, and finally, solving and post-processing many micro-scale models in parallel (Figure 1).

### Macro-scale Model

**Figure 2:**
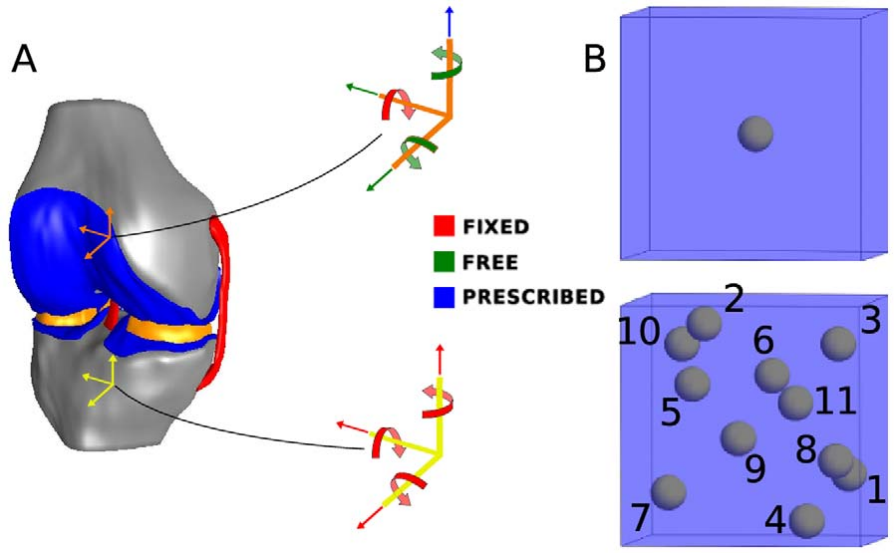
Macro and micro-scale models used in study. **A**. A model of the tibiofemoral joint was employed at the macro-scale. Boundary conditions were prescribed for the bones, modeled as rigid bodies, to approximate compressive loading of the joint. The tibia was fixed in space. Femoral compression was prescribed at fixed flexion with other degrees of freedom unconstrained. **B**. The single (top) and 11 cell (bottom) models. The single cell model was the popular scenario considered in previous studies [39], while the 11 cell model better represented the cell densities observed *in situ* for the middle layer of articular cartilage in the knee [40].

**Figure 3:**
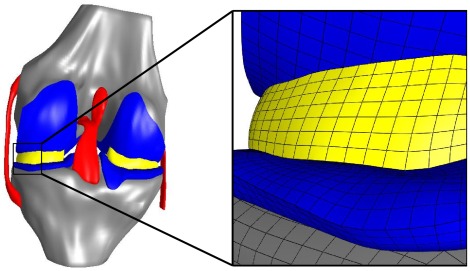
A finite element model of the tibiofemoral joint with representation of ACL, PCL, MCL, LCL, tibial and femoral cartilage, lateral and medial menisci, femur, and tibia. Soft tissue structures were discretized with 56433 linear hexahedral finite elements with an average edge length of approximately 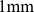. The bones were discretized with 25220 quadrilateral shell elements with an assigned thickness of 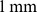. An enlarged model region is shown on the right to illustrate the mesh resolution. The femoral and tibial cartilage each had 3 hexahedral elements through their thickness.

A free and open access finite element representation of the tibiofemoral joint with modified material properties and boundary conditions (Open Knee version 1.0.0) was used to obtain a macro-scale solution [28]. The tibia, femur, medial collateral ligament (MCL), lateral collateral ligament (LCL), anterior cruciate ligament (ACL), posterior cruciate ligament (PCL), medial and lateral menisci, and femoral and tibial cartilage were represented (Figure 2A). All soft tissue structures were discretized with 56433 linear hexahedral (8 node) finite elements (Figure 3) and assigned hyperelastic material properties valid for finite strain, based on literature values (Table 1), while the bones were discretized with 25220 quadrilateral shell elements with a thickness of 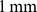 and assumed to be rigid.

**Table 1:** Material parameters for articular cartilage [30], ligaments [31]–[33], and meniscus [34]–[46].

	*ρ*	c_1_	c_2_	c_3_	c_4_	c_5_	K	λ^*^
**Cartilage**	**1.5e3**	**1.6892**	**0**	**-**	**-**	**-**	**83.3333**	**-**
**ACL**	**1.5e3**	**1.95**	**0**	**0.0139**	**116.22**	**535.039**	**73.2**	**1.046**
**PCL**	**1.5e3**	**3.25**	**0**	**0.1196**	**87.178**	**431.063**	**122**	**1.035**
**MCL**	**1.5e3**	**1.44**	**0**	**0.57**	**48**	**467.1**	**397**	**1.063**
**LCL**	**1.5e3**	**1.44**	**0**	**0.57**	**48**	**467.1**	**397**	**1.063**
**Meniscus**	**1.5e3**	**4.6115**	**0**	**0.12**	**150**	**400**	**227.5**	**1.02**

Units: 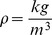; 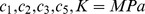; 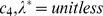.

An uncoupled isotropic Mooney-Rivlin constitutive model was used to define articular cartilage [29]. Model parameters were assigned to agree with literature values for instantaneous (fast-loading) elastic modulus (10 MPa) [30] and to simulate nearly incompressible (Poisson’s ratio of 0.48) behavior (Table 1). Ligaments [31]–[33] and menisci [34]–[36] were modeled as transversely isotropic with a Mooney-Rivlin ground substance and a piecewise non-linear fiber term [37] (Table 1).

Frictionless contact was defined between all soft tissue structures which may interface. The tibia was fixed in space for the entire solution time, while the femur was prescribed a distal (compressive) displacement of 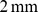 linearly ramped from 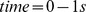 with flexion fixed. Displacement, rather than force, was prescribed as this was a better numerically conditioned problem. All other femoral degrees of freedom were free, allowing the femoral trajectory to be decided by the combined effects of soft tissue structures. Implicit dynamic analysis was conducted using FEBio version 1.4 [38]. A dynamic model was used to exploit the lumped mass matrix to condition the stiffness matrix at each non-linear time step and aid in model convergence. The deformed nodal positions were the outputs of the macro-scale model to be processed for multi-scale coupling.

### Joint-scale to Cell-scale Mechanical Coupling

Mechanical coupling between spatial scales was achieved by passing deformation gradients occurring at the macro-scale to the micro-scale. The deformation gradient is a second-order tensor that maps a position vector from the undeformed state, 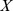, to a deformed state, 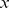. The undeformed and deformed nodal positions from the macro-scale model were used to calculate the deformation gradients occurring at element centroids for user-specified element sets at user-specified simulation times. For the current study, the results reported were for the tibial and femoral cartilage at a simulation time of 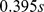. At this converged time point in the simulation, a compressive force of approximately 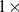 specimen body weight (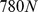) occurred. The last converged time step in the macro-scale model was 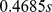, at which a compressive force of 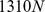 occurred. This solution set therefore allows for the potential consideration of up to nearly 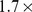 specimen body weight.

Calculating the deformation gradient from the deformed nodal positions made use of the isoparametric formulation technique often employed in FEA. Briefly, this formulation maps an element in the global space to an element of idealized geometry where calculations can be performed in a simplified manner. To calculate the deformation gradient at the element centroids the following equations were evaluated.
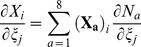
(1)

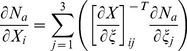
(2)

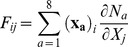
(3)


where, 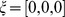 was the centroid position vector in the idealized geometry space, 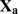 were the undeformed nodal position vectors, 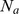 were the trilinear nodal shape functions, 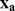 were the deformed nodal position vectors, 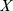 was the position vector of the element in the global space corresponding to the point in the idealized space, 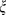, and 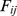 was the deformation gradient. The deformation gradients calculated from Equation 3 were used to prescribe boundary conditions for each micro-scale model.

### Micro-scale Models

Micro-scale model input decks for elements that experienced deformation (characterized by having a deformation gradient that varied from the identity tensor by greater than 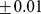 in any component) were generated. A total of 7882 elements in the tibial and femoral cartilage were above this threshold and warranted model generation. Two micro-scale cases were considered: a single cell and an 11 cell configuration (Figure 2B). In the single cell case, a spherical cell (of radius 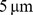) surrounded by a pericellular matrix (PCM) (of thickness 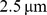) located at the centroid of a 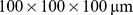 block of extracellular matrix (ECM), similar to previous studies [39], was considered. For the 11 cell case, cells and PCMs of the same dimension as the single cell configuration were randomly positioned in the ECM block, with the constraint that all chondrons (chondrocyte + PCM) were separated by at least 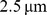 (equivalent to pericellular matrix thickness) and were at least 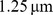 from the outer boundary. Eleven cells corresponded to the mean number of cells that occur in a block of middle layer tibiofemoral articular cartilage of this size [40]. Materials were defined with an uncoupled Mooney-Rivlin constitutive model with values from [41] adjusted to approximately satisfy mechanical consistency across spatial scales (Table 2). This was achieved by assigning the ECM, which comprises 99.5% and 94.2% of the construct volume for the single and 11 cell cases, respectively, identical properties to that of the cartilage in the macro-scale model. Previous studies have reported the PCM may have a strain amplification/attenuation effect on the cell due to the stiffness mismatch between ECM, PCM, and cell [42]–[44]. Therefore, the cell and PCM properties were scaled to maintain the same ratios as values reported in [41].

**Table 2:** Material parameters for micro-scale model [41].

	c_1_	c_2_	K
**ECM**	**1.6892**	**0**	**83.3333**
**PCM**	**0.6838**	**0**	**1.0570**
**Cell**	**0.0405**	**0**	**1.9980**

Units: 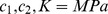.

The nodes on the six faces of the ECM block were prescribed displacement boundary conditions, 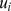, derived from the application of the macro-scale element deformation gradients, 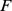, to their undeformed position vectors, 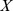 (Equation 4).
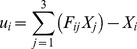
(4)


**Figure 4:**
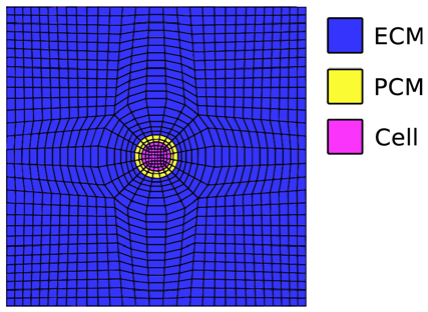
A cross-section of the 25889 finite element mesh of the single cell micro-scale case, which was determined to be mesh-converged. The extracellular matrix (ECM), pericellular matrix (PCM), and chondrocyte (Cell) were discretized with linear hexahedral elements. The chondrocyte had 9 elements across its diameter and the pericellular matrix had 2 elements through its thickness. For the eleven cell case, the chondrocyte and pericellular matrix had the same mesh densities.

Micro-scale models were solved using implicit static analysis in FEBio version 1.4. For verification, a mesh convergence study was conducted for the single cell geometry subjected to −30% nominal strain in the z-dimension with free lateral (x- and y-dimensions) expansion boundary conditions (in other words, unconfined uniaxial loading). The maximum effective strain and maximum shear strain that occurred in the model changed by 3.6% and 1.9%, respectively, when the mesh was increased from 15168 to 25889 linear hexahedral elements. Therefore, the 25889 element model (Figure 4) was assumed to be mesh-converged. The 77880 hexahedral element model of the 11 cell geometry was assumed to be mesh-converged because the cells and PCMs had the same mesh densities as the converged single cell case, 9 elements across the cell diameter and 2 elements through the PCM thickness, and the ECM had higher density.

### Micro-scale Model Deformation Analysis

To quantify the deformation of cells in the micro-scale models, the volume-averaged effective (von Mises) strain, effective stress, and maximum shear strain, as well as, the initial and deformed aspect ratios were calculated for the cell, by operations on the stress and strain tensors generated by the model simulations for each element. Cell level deformation metrics required volume-averaging across the cell. Undeformed (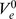) and deformed (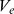) element volumes within the cells were used for volume-averaging. The undeformed element volumes were determined from nodal positions read from the micro-scale mesh definition file. Likewise, the deformed nodal positions, the result of the undeformed nodal positions plus the nodal displacement vector, were used to calculate the deformed element volumes. The mesh definition file also contained element sets, which were used to define micro-scale sub-regions e.g. the cell(s). The volume of each element, 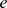, in the mesh was calculated with Equation 5,
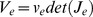
(5)where, 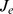 were the Jacobian matrices evaluated at each element centroid (the determinants of which were the volume ratios) and 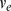 were the isoparametric element volumes. For the hexahedral case, the isoparametric element was defined as a 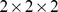 cube (with the same units as the model units), thus 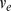 was always 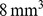. The Jacobian matrix maps the isoparametric space 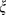 to the global or model space, 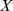. Element volume was calculated in this manner for both the deformed cases as well as the undeformed case.

#### Change in cell volume

Volumetric strain for each cell was calculated by Equation 6.
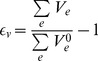
(6)


where, 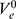 was the undeformed volume of element 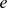 in the cell.

#### Change in cellular aspect ratio

The shape(s) of the cell(s) was calculated by assembling the moment of inertia tensor for the finite elements contained in the cellular subsets. With the assumption of unit density, the moment of inertia tensor, 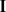, was calculated with Equation 7,
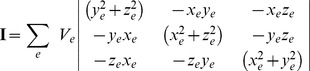
(7)where, 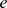 was each finite element belonging to a cell set, 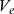 was the element volume, and 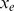, 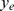, and 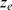 were the components of the element centroid position vector.

The eigenvalues, 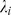 in descending order, of this tensor correspond to the principal moments of inertia which can be related to the length of the axes of the ellipsoid of best fit for the object’s shape, 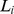, via Equation 8 [45].
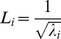
(8)


Three aspect ratios were measured: 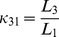, 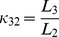, and 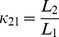, the major-minor, major-middle, and middle-minor axes ratios, respectively. The change in cellular aspect ratio was then taken as the difference between the undeformed and deformed aspect ratios.

#### Stress and strain metrics

The effective stress and strain were positive definite scalar values calculated from the Cauchy stress and Green-Lagrange strain tensors via Equation 9,
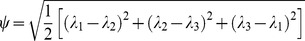
(9)where, 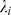 are the eigenvalues of the stress or strain tensor in descending order. These eigenvalues correspond to the principal stresses or strains.

Likewise, maximum shear strain, calculated by Equation 10, was also positive definite, and scalar.
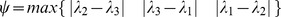
(10)


Since these metrics were positive definite scalars, volume-averaging was valid for a set of finite elements. This was performed for the cell(s), PCM, and ECM as follows:
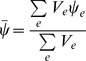
(11)where, 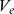 and 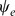 were the deformed volume and stress or strain metrics for each finite element contained in the respective set.

### Parallelization

Since all micro-scale models were independent; generation, solution, and analysis of them could be performed easily on a distributed memory computational platform. All micro-scale computational work for this study was performed on Ohio Supercomputer Center’s Glenn Cluster (http://www.osc.edu), which provides up to 9572 compute cores, ranging in frequencies of 2.4–2.6 GHz, offering a peak performance of more than 75 teraflops. A collection of Python and shell scripts were used to generate, solve with FEBio, and analyze a unique micro-scale model for each finite element experiencing deformation in the middle layer of tibial and femoral cartilage. 7882 model generation, solution, and analysis processes were divided between 101 compute threads, with 78 process sets carried out in serial on 100 threads and 82 on the 101st thread. The pipeline completed in a wall-clock time of approximately 2 hours for the single cell case and 19 hours for the 11 cell case. These corresponded to CPU times of approximately 8.4 and 72.3 days, respectively; demonstrating the importance of parallelization.

## Results

**Figure 5:**
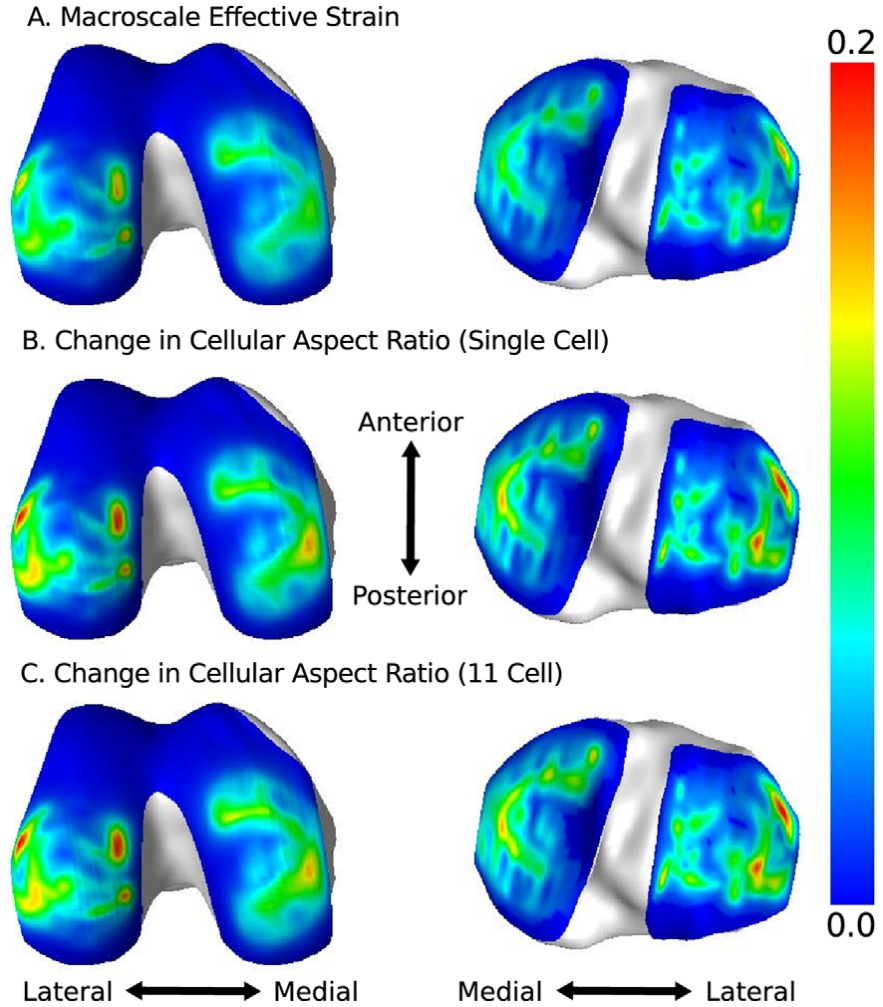
Fringe plots showing macro-scale and micro-scale deformation metric regional distributions in the middle layer of cartilage of the femur (left) and tibia (right). **A**. The effective strain which occurred at the macro-scale. **B**. The change in cellular aspect ratio (major-minor) calculated for the single cell model. **C**. The maximum change in cellular aspect ratio (major-minor) experienced by a cell in the 11 cell model.

The FEA solution of the tibiofemoral joint model under 1× body weight compression (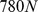), resulted in strain distributions with concentrations under the menisci-cartilage interfaces for both tibial and femoral cartilage (Figure 5A). The cell(s) in both the single cell and 11 cell models experienced amplified deformation when compared to the macro-scale finite element deformation which drove the mechanics at the micro-scale model exterior. This was expected due to the large mismatch in micro-scale component material properties, i.e. cells being softer than their surrounding medium, resulting in inhomogeneous deformation occurring in the interior. Although the magnitudes were different, the regional distribution of deformation was similar to that which occurred at the macro-scale (Figure 5B and 5C).

**Figure 6:**
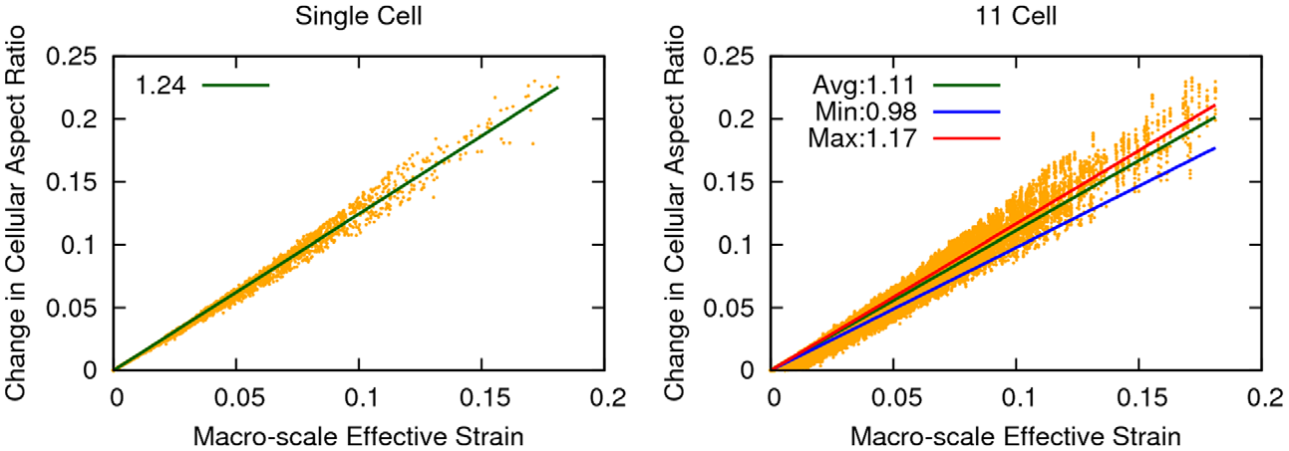
The change in cellular aspect ratio measured in each micro-scale model for the single cell (left) and 11 cell (right) cases was plotted as a function of macro-scale effective strain occurring at the corresponding finite element. The solid lines were linear least-squares regressions performed with the requirement that the lines passed through the origin. The slopes of each of these lines were provided in the legend. In the 11 cell case, the line labeled “Max” considered only data from the cell which experienced the greatest change in aspect ratio. Likewise, the line labeled “Min” was the cell which experienced the least change in aspect ratio, and the “Avg” was the average of the linear regressions for all 11 cells. These slopes indicated the distribution of the 11 cell change in aspect ratios was skewed left.

The change in cellular aspect ratio, 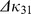, was linearly proportional to the macro-scale effective strain for both the single cell and 11 cell cases, with the 11 cell case consistently lower for all cells than the single cell case (Figure 6). In both configurations, the data spread increased as the corresponding macro-scale deformation increased, with the effect magnified in the 11 cell case. This behavior can be quantified by considering the sum of squares of residuals (SSR) attained for the linear fits. A greater SSR value indicates a larger spread (Table 3).

**Table 3:** Linear regression slopes and sum of squared residuals of single and 11 cell cases for change in cellular aspect ratio vs macro-scale effective strain.

	Single Cell	11 CellAverage	11 CellMinimum	11 CellMaximum
Slope	1.2429	1.1119	0.9765	1.1652
SSR	0.0513	0.1718	0.0949	0.1986

**Figure 7:**
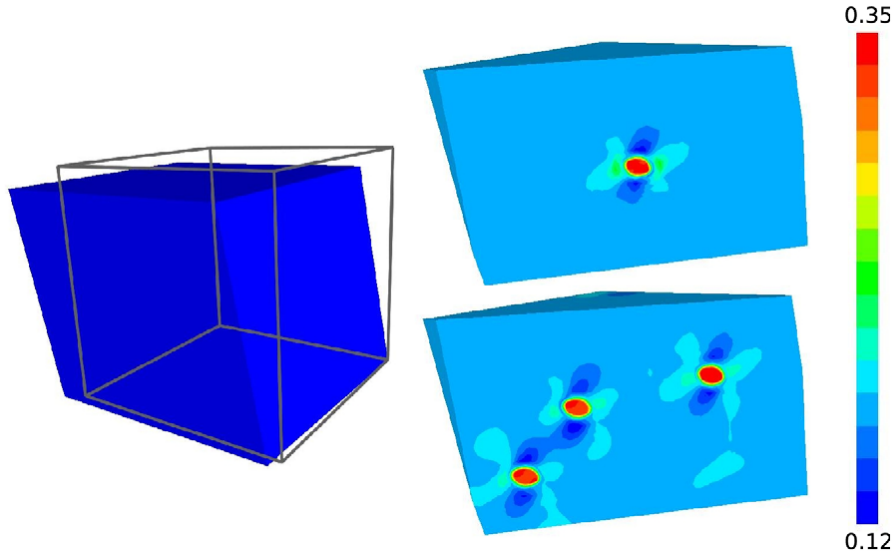
An example of the internal effective strain distributions observed in a single (top right) and 11 cell (bottom right) micro-scale solution for the same deformation gradient passed from the macro-scale (left). A macro-scale finite element experienced a combined loading deformation gradient which resulted in a shape change from the gray cube to the blue hexahedron shown on the left. The single and 11 cell models were deformed in the same way with the resulting effective strain distributions in a cross-section of the model shown on the right. While the cells experienced qualitatively similar strains in both cases, the strain in the ECM around the cells in the 11 cell model differed from the single cell in regions of close cellular proximity. See Table 4 for cell-specific quantitative results.

Regardless of the presentation of the results in the form of linear relationships between macro-scale and micro-scale, the micro-scale solutions had an inhomogeneous strain distribution internally. This was of particular interest when comparing single and 11 cell configurations, where cellular proximity altered distribution in the 11 cell models (Figure 7). A more detailed look to cell deformation metrics for this specific case of macroscopic deformation also illustrated that magnitudes of individual cell deformations may differ, potentially based on their location (Table 4). As described in the methods, deformation metrics were also obtained for the ECM and PCM. These results, although not presented, may have utility when investigating matrix damage mechanisms.

**Table 4:** Chondrocyte deformation metric set for a single and eleven cell model with boundary conditions prescribed via a specific deformation gradient passed from the macro-scale.

Cell ID	Δ Aspect Ratio	Volumetric Strain	Effective Strain	Maximum Shear Strain	Effective Stress
Single	0.226	−0.130	0.341	0.197	1.028
Cell 1	0.215	−0.108	0.334	0.193	0.301
Cell 2	0.200	−0.100	0.314	0.181	0.270
Cell 3	0.202	−0.100	0.317	0.183	0.272
Cell 4	0.196	−0.096	0.309	0.178	0.276
Cell 5	0.224	−0.113	0.345	0.199	0.311
Cell 6	0.220	−0.111	0.339	0.196	0.306
Cell 7	0.218	−0.110	0.336	0.193	0.302
Cell 8	0.216	−0.109	0.334	0.193	0.300
Cell 9	0.220	−0.111	0.338	0.195	0.304
Cell 10	0.212	−0.107	0.329	0.190	0.293
Cell 11	0.216	−0.109	0.334	0.193	0.301
Mean 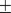 SD	0.213 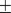 0.009	−0.107 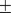 0.006	0.330 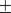 0.011	0.190 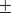 0.007	0.294 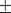 0.015

See Figure 7 for visualization and Figure 2B for the location of individual cells within the eleven cell model.

## Discussion

The method employed in this study was a preliminary step toward the realization of relating joint level mechanics to the mechanics of the cellular environment. While a similar method has been employed for simplified tissue geometries at a small subset of spatial coordinates [46], to the best of the authors’ knowledge, this was the first study which investigated large tissue regions, and considered anatomically-based geometry (both at macro- and micro-scales). This provided coarse insight into how cells may be deforming *in situ*.

Some notable observations were made in comparison of single and 11 cell micro-scale models. In both cases, the deformations determined at the macro-scale, when applied to the micro-scale, resulted in amplified deformations of the cell(s). This behavior was expected, because the chondrocytes were much softer than the surrounding ECM. However, every cell in the 11 cell model experienced smaller deformations for a given macro-scale deformation than the single cell model did. If one considers a simple 1D analogy of springs in series to model the ECM-chondron composites, the addition of more soft springs, as is the case in the 11 cell model, would result in less displacement occurring in each of the soft springs when displacement is prescribed at the series ends. Extending this to 3D supports the model results. This strain-shielding trend in the 11 cell models may suggest that the single cell case provides an upper bound for the observed macro-micro deformation amplification. Cells within the 11 cell models also experienced different deformation from one another. Although the cell positions were assigned randomly for this model without input from histological observation other than cell count, it is conceivable that, in reality, cells within the middle layer of cartilage do not experience the same mechanics, and the resolution of this variance may be at the order of several microns. The proximity of chondrocytes to each other may have dictated their mechanics. While an analysis was not specifically conducted to explore the relationship between cell-to-cell distance and intercellular mechanical interaction; this study, through macro-to-micro response metrics (Figure 6), supported by a comparison of single and 11 cell model results for a specific macroscopic deformation (Figure 7 and Table 4), indicates that such an influence may exist. This is certainly an important issue to address in the future where evaluation of single cell mechanics within a large group may be a necessity.

In spite of using simplified geometries (at the micro-scale) and constitutive models (i.e. non-linear, isotropic elastic rather than anisotropic, poroelastic), this study presented opportunities to make several observations from a mechano-biological perspective. Elongation of cells of 10% has been shown to induce catabolic processes in SW1353 chondrocyte-like cells, while 5% was not shown to have an effect [47]. These elongations correspond to aspect ratio changes (major-minor) of approximately 0.154 and 0.024 respectively. Given the low femoral compressive force of 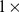 body weight prescribed in this study, in comparison to, the 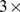 body weight or greater joint distal forces typically observed during walking [48], one would not expect cellular deformations to occur which may induce catabolic processes, yet changes in aspect ratios greater than 0.154 were observed. This indicated a limitation in the modeling at the macro-scale, micro-scale, or likely both, potentially related to the suitability of macro- and/or micro-scale material representations.

**Figure 8:**
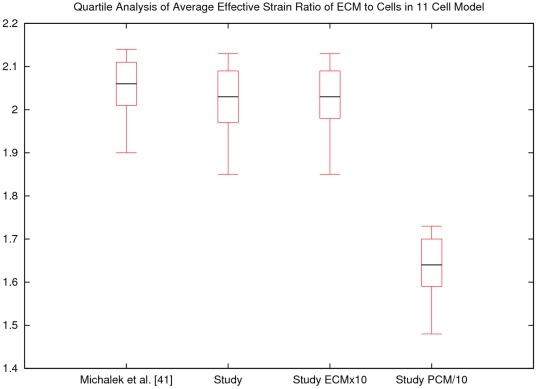
The quartile analyses of the ratios of average cellular effective strain to extracellular matrix (ECM) effective strain for the four cases considered in the material sensitivity study of the 11 cell model are shown above. In agreement with [41], scaling of material properties while maintaining ratio (Study and Study ×10) does not strongly impact chondrocyte deformation, but modifying the stiffness ratio between micro-scale components, i.e. by decreasing pericellular matrix stiffness by tenfold (Study PCM/10), does.

Modeling the material behavior of the macro-scale cartilage as too soft would be an obvious source of over-prediction of deformation. If this is the case in this study, the predicted macro-scale cartilage deformations, which would be necessary to equilibrate the desired level of joint loading, would be larger than expected. This will in turn result in higher chondrocyte deformations. Likewise, adjusting the PCM and cell stiffnesses will also have strong influence on the cellular deformation. The material properties assigned to the ECM, PCM, and cell were taken from a numerical study that optimized the PCM modulus to agree with experimental observation. The optimized PCM modulus found in this study resulted in a 2.11 ratio (cell/ECM) of effective strain, which varied from the experimentally observed ratio by 0.5% [41]. For the current study, the optimized material properties reported by [41] were scaled while maintaining the same ratios, and the case of multiple cells was considered. To assess potential sources of error due to this scaling, a simple sensitivity study was conducted in which the eleven cell model was subjected to a nominal compressive strain of 10% with volume-preserving lateral expansion for substantially different ECM properties: 1) 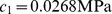, 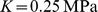 [41], 2) 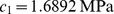, 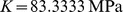 [reported study], 3) 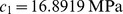, 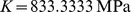 [reported study × 10]) while maintaining equivalent stiffness ratios between components. Additionally, a case in which the PCM stiffness reported in Table 2 was reduced by a factor of 10, 4) 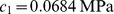, 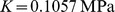, while the other components were unchanged, was considered. The quartile analysis of the ratios of the average effective strain occurring in the ECM vs that occurring in each cell for these four cases are presented in Figure 8. The scaling of the material properties from those reported in [41] to those used in this study resulted in small changes in cellular deformation, indicating the scaling approach did not necessarily influence the amplification of macro-scale strains on the cells. Likewise, scaling the material properties by an additional factor of 10, resulted in little change. In contrast, reducing the PCM stiffness by tenfold resulted in a decrease in cellular effective strain, in agreement with the trends observed in [41].

For informative results, computational models should ideally employ anatomical geometry, realistic constitutive representations, and physiological joint loading. In addition, simulation results should be confirmed by comparisons against experimental studies. This study provided an adequate representation of anatomy at both joint and cell levels. The use of constitutive models that capture the anisotropic, lamellar, and poroelastic behavior of cartilage at the macro-scale would likely alter the strain distribution and magnitude within the tissue. While these complicated material models have commonly been employed at the micro-scale [23], their advent was quite recent at the joint scale. These state-of-the-art joint models incur high computational cost, and are currently only capable of simulating loads smaller than those observed *in vivo* [49], and also lower than forces employed in this study. In addition to material assumptions, the loading conditions utilized in this study, while large in magnitudes, were not necessarily physiological, i.e. representative of gait. Joint scale models driven by physiological dynamics exist, but these must employ simplified material models, like those used in this study, and revert to explicit time integration for speed and robustness [50]. The highly non-linear behavior of cartilage, when modeled accurately, may be better suited for implicit time integration and the non-linear solution convergence checks it bestows. Currently, even at the single spatial scale of joints, modeling and simulation studies employing both physiologically accurate constitutive behavior and lifelike loading do not seem to be available.

**Figure 9:**
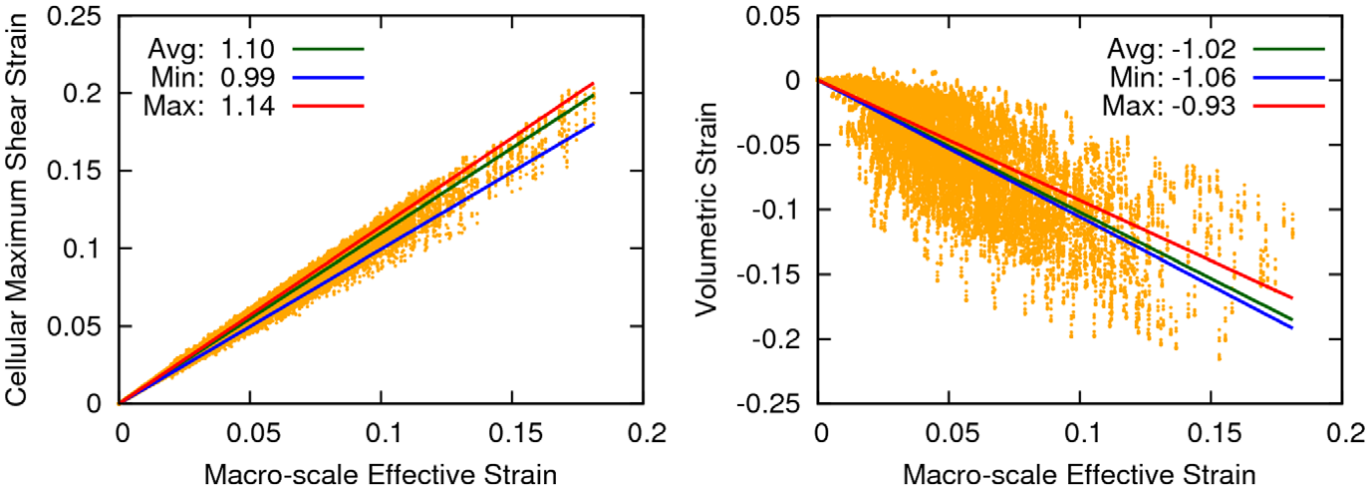
The volume-averaged maximum shear strain (left) and volumetric strain (right) for the 11 cell model plotted against the macro-scale effective strain. Deformation metrics such as those shown here, may have additional implications to understand cellular damage mechanisms as well as the onset of mechano-biological function, which change in aspect ratio may not provide. For example, pure cell dilation will result in a volumetric strain, but not a change in aspect ratio. The large bandwidth of the volumetric strain also suggests that a direct linear relationship between the macro- and micro-scales may not be acceptable for all characterizations of deformation.

As in any modeling study, verification and validation are necessary. The micro-scale models were verified through a mesh convergence study, but were not validated against an experimental study. In contrast, a mesh convergence study was not performed on the macro-scale model. However, a previous study found tibial and femoral cartilage modeled with elastic material properties converged with an approximate linear hexahedral element edge length of 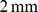 [51]. Therefore, the cartilage mesh used in this study, with an approximate element edge length of 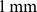, was assumed sufficient for elastic analysis. Adequate validation of biological structures at any scale; joint, tissue, or cellular, presents substantial challenges. In addition, the further complication of multi-scale coupling exacerbates the difficulty. The macro-scale model was weakly validated through assessing its ability to reproduce experimentally-observed joint kinematics [52], but this level of validation was not ultimately sufficient to the aspects of this study which were dependent on the strain distribution within the tissue rather than joint kinematics. Contact pressure, which can better reflect the internal strain state, is an obtainable measure [53], but in addition to added experimental difficulty, the introduction of measurement devices may modify the mechanics of the joint. Predictions of large chondrocyte deformations under one body weight suggests that further validation of the multi-scale modeling strategy may be necessary. While a direct validation to confirm chondrocyte deformations in the human knee, under physiological loading, may not be possible; recent animal studies may provide possible avenues to establish confidence in the multi-scale modeling approach presented in this study. For example, a recent application of multi-photon confocal microscopy quantified chondrocyte deformation *in situ* while an intact joint of a mouse was subjected to physiological muscle loading [22].

Change in aspect ratio has often been employed as the metric to evaluate deformation in experimentation [45]. Nonetheless, other variables of interest, such as those related to shear and volumetric strains, may have significant value to understand cellular damage mechanisms as well as thresholds of mechano-biological function. This modeling pipeline has the capacity to summarize such variables, as illustrated by Figure 9, showing cellular volumetric strain and maximum shear strain exhibited by cells of the eleven cell model.

This study also provided a large database of input-output relationships between macro-scale and micro-scale mechanics. The slopes obtained from linear regression analysis of this data provide a direct proportionality constant to relate mechanics obtained at the macro-scale to the deformation the chondrocytes may experience, with the uncertainty in that relationship quantifiable by the residuals of the fit. In addition, this database contained loading states which were a direct result of joint mechanics. While a similar database may be obtained with either idealized interval stepping through a series of deformation gradients or through the solution of a stochastic set of deformation gradient applied boundary conditions, the former may not capture all loading states which may possibly occur, while the latter may introduce states which may not occur *in situ*. This database was specific to the joint-level case of vertical compression of 1× body weight. It could be repeated for another joint scenario i.e. stair-climbing, to obtain another activity-specific database. The collection of these databases can provide a widely applicable surrogate model, i.e. analogous to approaches used in coupling of movement and tissue deformation simulations [54], to efficiently relate macro-micro scale mechanics without the need to perform micro-scale analyses.

**Figure 10:**
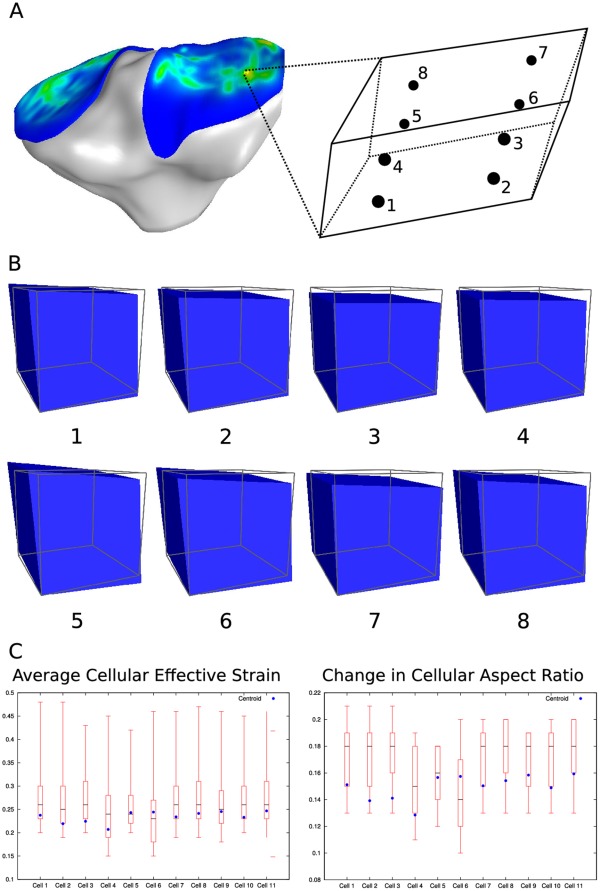
To investigate the potential error due to sampling of the deformation gradient at element centroids, 11 cell micro-scale models were solved for the eight Gaussian integration points of the macro-scale element determined to have the highest variation in deformation gradient. **A**. Deformation gradients were calculated at the eight integration points of macro-scale element ID 20585. **B**. The micro-scale model boundary conditions, assigned for each integration point and identified in the image sequence, resulted in a range of deformations. **C**. The variation in deformation gradient associated with integration point sampling resulted in a variation in cellular deformation metrics. The quartile analysis of the resulting volume-averaged cellular effective strain and change in cellular aspect ratio is shown above with the value determined from the analysis performed at the macro-scale element centroid overlaid.

The pipeline developed was proven to be highly scalable allowing for reasonably fast solution of the multi-scale problem. Since iterative multi-scale communications were not required during the processing and analysis stages of the many micro-scale simulations, the network overhead only involved movement of files to and from each parallel node at the beginning and end of the generation, solution, and analysis process. It was therefore, fair to hypothesize that this method, although tested on 101 parallel threads, should scale well to thousands of threads. This scalability will prove invaluable when more complex micro-scale models or a larger array of macro-scale elements are considered.

Only passing the deformation gradient at the macro-scale finite element centroids potentially introduced error. A hexahedral finite element can deform such that the deformation gradient is non-uniform across its volume. Depending on the mode of loading, this variation could be high, and sampling only at the macro-scale element centroids may alias the deformation occurring on the continuum discretized by the finite elements. To assess this sampling error, the deformation gradients were calculated at each of the 8 Gaussian integration points in the macro-scale finite elements. Deformation gradient variation on each element was then characterized by taking the difference of every unique combination of the 8 deformation gradient tensors, summing the absolute value of the components of the tensor resulting from each difference to get a scalar residual value, and then taking the highest of these 8 scalar residuals and using that as a metric to describe the deformation gradient variation on the particular element. The deformation gradients calculated at the element with the highest variation metric, element ID 20585 with a residual of 0.317, (Figure 10A) were used to assign boundary conditions to eight different eleven cell micro-scale models (Figure 10B). The deformation modes ranged from compression dominated, e.g. integration point 3, to shear dominated e.g. integration point 5. The cellular deformation metrics were calculated from the results of these 8 models for each chondrocyte and a quartile analysis for each cell was performed (Figure 10C). The range in the resulting cellular deformation metrics is high, e.g. 30% in effective strain. While this is the worst case element, it certainly illustrates the sensitivity to spatial sampling. To reduce this error, one can either increase the spatial sampling resolution, e.g. sample at integration points rather than centroids, or refine the macro-scale mesh. Both cases can easily be implemented in this approach, with the acceptance of added computational cost.

Prescribing boundary conditions based on the deformation gradient modeled the finite strain as a first order Taylor series. For problems that experience a highly non-uniform deformation gradient, this may not be an adequate approximation. Including an additional term in the series would capture the gradient of the deformation gradient, and therefore, include information about how the deformation gradient varies spatially [26]. From a temporal perspective, the macro-scale deformation information was only passed to the micro-scale at a single instant in time. The time history of the macro-scale element deformation prior to this instant was not communicated. While this is not relevant in an elastic analysis, including this information will be necessary when considering rate-dependent phenomena such as poro- or visco-elasticity.

The mechanical consistency across spatial scales was satisfied in a weak sense through the use of the same constitutive models at the macro and micro-scales. The PCM(s) and cell(s), although much softer were negligible in volume compared to the ECM. The error associated with this assumption would not be present when using a computational homogenization approach in which the macro-scale material behavior resulted from the micro-scale constitutive models(s). However, the computational scalability advantage provided by the employed method was considered to outweigh the weaknesses in mechanical consistency satisfaction. With advances in shared-memory platform technology, the computational homogenization approach can be revisited in the future.

Due to the infancy of the presented research, a vast array of extensions can be made to the approach in the future. Considering more complex material behaviors at the macro and micro-scales should not only improve the predictivity of the modeling pipeline, but will also permit more accurate investigation of mechano-biological processes. Regarding predictivity, the crude linear relationship, presented in this study for elastic deformations of chondrocytes and joint loading, may take more complicated forms with more accurate constitutive modeling. Extension to a poroelastic model will supply a fluid flux vector field providing insights into bulk fluid flow for transport of nutrients, wastes, and signaling agents. It has been shown that chondrocytes respond to fluid shear stress at the cellular membrane [55], but not to the hydrostatic pressure acting normal to it [56], [57]. The fluid flux vector field returned by a poroelastic model will allow for quantitative measures of these transverse and perpendicular quantities. Likewise, the incorporation of fibrous structures into the material models of the micro-scale constituents will also have mechano-biological implications. Such additions will provide a coarse means to model, for example, cytoskeleton fibril interaction with integrin binding sites [4] and stretch-induced ion channel activation [56], [57]. An additional coupling to mechano-biological models may also be possible, providing a link between daily activity and phenomena such as matrix remodeling, cell migration, and apoptosis [58]. Ultimately, this will aid in the understanding of the pathological and/or age-related evolution of cartilage anatomy, physiology, and mechanics.
